# Predictors of High Obesity in Rural Nicaragua: A Cross-Sectional Study

**DOI:** 10.3390/ijerph22050660

**Published:** 2025-04-22

**Authors:** Karen Herrera, Milena Marquina de Reyes, Thankam Sunil

**Affiliations:** 1 Predicate Labs-CIES-UNAN Nicaragua, Managua 14172, Nicaragua; 2 The Specialized Health Research Center, Evangelical University of El Salvador, San Salvador 1101, El Salvador; 3 Department of Public Health, University of Tennessee, Knoxville, TN 37996, USA

**Keywords:** obesity, rural, behavioral factors, food

## Abstract

Chronic disease prevalence continues to increase in low- and middle-income countries, and the countries in the Central American region are no exception. Recent reports have shown that women are particularly at higher risk for being obese or overweight in several countries in Central America, including Nicaragua. In the present study, we conducted a cross-sectional analysis of a sample of respondents (*n* = 200) who were aged 30 years and over and living in rural communities in Nicaragua. This study’s results show that a higher percentage of the respondents reported their health as being fair or poor, and female respondents were found to have higher BMIs compared to their male counterparts (*p* < 0.05). While previous studies have noted that, traditionally, the body mass index (BMI) has been the most widely used measure to assess overweight prevalence in populations and to evaluate individual health risks, this study used the waist–hip ratio to measure the prevalence of obesity in adults. In recent years, the central obesity indicators, primarily waist circumference and the waist-to-hip ratio, have been recognized as more accurate at describing body fat distribution compared to the BMI. These measures have also been found to have a stronger association with morbidity and mortality. Behavioral factors, such as vegetable consumption and hours of sleep, were found to be significant predictors of obesity/overweight among rural residents in Nicaragua. This study’s results highlight the need for targeted behavioral change interventions, including promoting the regular consumption of fruits and vegetables in the diets of rural residents.

## 1. Introduction

In recent years, low- and middle-income countries in the Americas have seen a concerning rise in obesity and overweight among adults [1] (WHO, 2014), a trend expected to continue in the coming decades [2]. For instance, in Mexico, the obesity rate (BMI > 30 kg/m^2^) increased from 24.2% in 2002 to 36.9% in 2022. Similar trends have been observed in Chile, where the rate rose from 20.5% in 2000 to 30.7% in 2022, and in other countries [3]. These increases have been particularly significant among women. In Mexico, the percentage of obese women rose from 29% in 2000 to 41% in 2022, while for men, it was 32.3% in 2022. Projections indicate that by 2030, overweight and obesity will affect 50% of males and 60% of females in Latin America [4].

In Nicaragua, the percentage of adult obesity has also been steadily increasing, with higher rates among women. The most recent report on obesity in Nicaragua showed that 32.1% of adult (aged 18 years and over) women and 20.6% of adult men are living with obesity. This is an increase from its prevalence in the year 2000, which was 20.3% and 10.6%, respectively. Nicaragua’s obesity prevalence is higher than the regional average of 30.7% for women, but is lower than the regional average of 22.8% for men. At the same time, diabetes is estimated to affect 12.5% of adult women and 10.5% of adult men [3].

The obesity epidemic shares common characteristics worldwide, with over 60% of individuals with excess weight residing in low- to middle-income countries. One of the main risk factors is urbanization, which is associated with a predominantly sedentary lifestyle and widespread access to high-calorie, low-nutrient “junk food” [4,5,6,7].

Chronic non-communicable diseases (NCDs) are the leading cause of death in the Americas, accounting for 81% of all fatalities. It is estimated that 240 million adults in the region live with at least one [7]. Overweight and obesity are now recognized as chronic non-communicable diseases influenced by genetic, social, and environmental factors, including lifestyle choices and socioeconomic determinants [8]. These conditions are considered a global and national public health epidemic, contributing to comorbidities, such as diabetes, ischemic heart disease, and certain types of cancer [9].

However, studies on obesity and its contributing factors in Nicaragua are limited. As reported in a previous study, there are no studies on Nicaragua that have looked at the factors affecting obesity and have been published in English [10] and, specifically, no studies investigating obesity predictors in rural areas, thus leaving a gap in understanding obesity in rural communities. This study aims to explore the risk-taking behaviors of individuals in rural Nicaragua and assess the impact of nutritional and physical activities on obesity.

## 2. Data and Methods

The data for this study were obtained from a cross-sectional survey conducted in the rural communities of Quebrada Honda, Nadayure, and Diriomito in the department of Masaya, Nicaragua. These three communities were selected randomly, and represent rural characteristics of the population. Data collection was carried out by family physicians familiar with the area who visited houses randomly. We selected 200 respondents aged 30 years and older who had no medically diagnosed chronic health conditions. Using G*Power with the following parameters—effect size d = 0.5, alpha err prob = 0.05, Power = 0.8, and Allocation ratio N2/N1 = 1—we determined that a total sample of 192 participants was needed [11]. The survey questionnaire was an adapted version of the Behavioral Risk Factor Surveillance System (BRFSS) from the Centers for Disease Control and Prevention (CDC) [12]. It underwent validation to ensure its relevance, cultural appropriateness, and comprehensibility for rural populations. The instrument collected data on sociodemographic variables, such as age, marital status, education level, and occupation, along with self-reported perceptions of physical and mental health over the past 30 days. It also assessed dietary habits, physical activity, and other health-related behaviors. Physical activity data were collected by asking the participants if they engaged in any type of physical activity, such as walking, running, aerobics, cycling, or exercising at home, with the aim of improving their health. If they reported yes, we asked for frequency of such activities.

Additionally, we collected weight data using a digital scale, ensuring patients wore minimal clothing for accuracy. Weight was recorded in kilograms, and the scale was calibrated daily before use. Height was measured using a stadiometer, with results recorded in centimeters. We calculated the Body Mass Index (BMI) using the following formula: weight (kg)/height (m)^2^. BMI categories were defined according to WHO as follows: Underweight: BMI < 18.5; normal weight: BMI 18.5–24.9; overweight: BMI 25–29.9; and obesity: BMI ≥ 30 [13].

Waist–hip ratio was used for obesity analysis since BMI does not consider body fat distribution and WHR does. BMI has an important limitation, since there are suggestions that the metabolic complications of obesity are more closely related to visceral adiposity than overall adiposity. Results from most cross-sectional studies have shown a stronger association of CVD risk factors with central obesity (WC or WHR or WSR, or all) than with general obesity (BMI) [14]. WHR was measured using a flexible measuring tape at the narrowest part, usually just above the belly button. Hip circumference was measured at the widest part of the hips. The waist-to-hip ratio (WHR) was calculated by dividing the waist circumference by the hip circumference, ensuring both measurements were in the same units. Waist–hip ratio classification followed WHO guidelines: ≥ 0.90 for men and ≥ 0.85 for women [15].

Fasting glycemia tests were performed by laboratory personnel, ensuring proper technique. The samples were immediately transported with the appropriate safety measures and temperature control to prevent any alterations. The processing of the samples was carried out promptly at a nearby laboratory with the proper certifications for such studies.

Blood pressure was measured using an aneroid sphygmomanometer, calibrated daily. It was ensured that the participants had fasted for at least 8 to 12 h before the test (only water was allowed). Additionally, they were asked whether they had consumed alcohol in the previous 24 h. Likewise, it was confirmed that they had not smoked or engaged in intense physical activity before the test, as these factors could have affected the results.

Although the research was not experimental, the ethical principles of the Helsinki Declaration for medical research involving human subjects were respected. This study’s protocol was approved by the Institutional Ethics Committee of the Military Hospital in Nicaragua with code SCEHM 2023-5.

Bivariate analysis with chi-square tests was conducted to understand the significant associations between variables. Two logistic regression analyses were performed to predict waist–hip ratio: model 1 adjusted for behavioral characteristics and self-reported health, and model 2 adjusted for behavioral characteristics, background variables, and self-reported health. The dependent variable was coded as 0 for normal or moderate waist–hip ratio and 1 for high waist–hip ratio. All analyses were conducted using SPSS statistics 29.0.2.

## 3. Results

Table 1 presents the background characteristics of the respondents. The median age of the respondents was found to be the age group 45–49, with the highest percentage of the respondents in the age group 30–34 (23.5 percent). Approximately half of the respondents reported a primary level of education.

When asked about their self-perceived health, more than two-thirds of the respondents (68 percent) reported their health as being either fair or poor; an overwhelming 41.5 percent of the respondents mentioned having 8 or more days with mental health problems. When asked if a health professional had ever diagnosed them with any health conditions, 28.5 percent reported having been diagnosed with high blood pressure.

In terms of risk-taking behaviors, a small percentage of the respondents reported smoking cigarettes (3.5 percent) or consuming alcohol (5.0 percent). On the other hand, when it came to protective behaviors, such as consuming vegetables, over two-thirds of the respondents reported never eating vegetables or salad. We also found that three-fourths of the respondents had had a routine medical checkup in the last year. The average number of hours of sleep per day was 7 h (see Table 2).

In addition, we collected information on several key clinical health measures. These measures included the BMI, blood pressure, glycemic levels, and waist–hip ratio (Table 2). In general, a BMI of 30+ was found among 41.5 percent of the respondents, over one-fourth of the respondents reported elevated or higher blood pressure, and a similar percentage of the respondents had above-normal glycemic levels, and 80 percent had a high waist–hip ratio.

The results from the bivariate analysis show that the females tended to have a significantly higher BMI compared to the males, and that lower levels of vegetable consumption led to significantly higher BMIs. These variables (gender and vegetable consumption), along with salad consumption, were found to be significantly associated with a high waist–hip ratio (Table 3).

Table 4 shows the odds of predicting a high waist–hip ratio for the respondents in this study. Two models are included in the table. Model 1 provides the odds ratio for the behavioral variables and self-reported health, and model 2 controls for the behavioral and self-reported health variables when predicting a high waist–hip ratio. The results show that after controlling for the background variables (age, gender, marital status, and educational level), the behavioral variables, such as hours of sleep and vegetable consumption, were significant predictors of the waist–hip ratio. That is, the odds of having a high waist–hip ratio were two times higher for those who reported more than 7 h of average sleep in the past week. It was also observed that the odds of having a high waist–hip ratio were lower among individuals who reported consuming vegetables/salads 2 or more times during the past week.

## 4. Discussion

Adult obesity in Nicaragua is a significant public health concern. According to the Global Nutrition Report, approximately 32.1% of adult women and 20.6% of adult men in Nicaragua are living with obesity. These rates indicate that obesity is more prevalent among women than men and exceed the regional averages for Latin America and the Caribbean, particularly for women.

Our study results have highlighted three key points. First, this study used the waist–hip ratio to measure the prevalence of obesity in adults, as opposed to the commonly used BMI measure. While previous studies have noted that, traditionally, the body mass index (BMI) has been the most widely used measure to assess overweight prevalence in populations and evaluate individual health risks, in recent years, central obesity indicators—primarily waist circumference and the waist-to-hip ratio—have been recognized as more accurate for describing body fat distribution compared to the BMI. These measures have also been found to have a stronger association with morbidity and mortality. Very few studies have used the waist–hip ratio to describe the prevalence of obesity and overweight in Latin American countries, particularly in Nicaragua [16,17].

Studies of developing countries in Asia and the United States have shown higher risks of cardiovascular disease with lower BMIs [18]. It has also been established that the waist–hip ratio is independently associated with mortality regardless of the BMI or comorbidities, and suggested that the waist–hip ratio might be a better indicator of mortality compared to the BMI, particularly for Hispanic and Latino populations. Despite these recommendations, studies on obesity and overweight in Nicaragua have often relied heavily on measuring the BMI. Thus, the results presented in this study are unique in using the waist–hip ratio and contribute further to our understanding of the growing public health concerns regarding overweight and obesity in Nicaragua. Future studies must consider using the waist–hip ratio as a viable measure for assessing obesity and overweight among adults and for developing prevention efforts addressing these issues.

Our study also found that a higher proportion of the respondents (more than 80%) reported a higher waist–hip ratio (Table 2), with this percentage being higher among the women than men. Approximately 85% of the females reported having a high waist–hip ratio, while 65% of the men reported the same Gender was significantly associated with the prediction of the waist–hip ratio, and the odds of predicting a higher waist–hip ratio were 3.58 times higher among the women compared to the men (*p* < 0.001) (Table 4). A higher prevalence of obesity and overweight among women has also been reported in previous studies. For example, Aschner [4] reported higher waist–hip ratios or obesity levels in females, and several national-level reports and studies from Latin American countries [4] have also reported higher levels of obesity among females. In addition to biological determinants, social and cultural factors play significant roles in predicting gender differences in obesity in Nicaragua. Economic deprivation may contribute to higher rates of obesity among women of low socioeconomic status due to their greater exposure to and dependence on high energy-dense foods. People who do not have physical or economic access to adequate food will seek to satisfy their energy needs through low-cost foods with low nutritional quality, which usually have high levels of saturated fats, monosaccharides, and energy intake [19].

Second, we learned that the number of residents living in rural communities who reported being in fair or poor health was very high in Nicaragua. A further analysis found that the respondents who reported their health as either fair or poor tended to be older (44 years and above), reported a higher waist–hip ratio, and had fewer average hours of sleep (less than 7 h per day). Behaviorally, these respondents also tended to work longer hours (9+ h on average per day) and consumed fewer or no vegetables. By knowing the background profile of respondents with higher waist–hip ratios, prevention efforts can be targeted to address behavioral challenges, including improving vegetable consumption and sleep quality.

The way individuals perceive their health is influenced by a complex set of factors, including environmental, cultural, and socioeconomic conditions. In the studied population, the majority of the respondents were women under the age of 50. Overall, their perception of their health was predominantly reported as fair or poor. Similar surveys have indicated that the disparities observed between men and women regarding medical consultations and health check-ups may be explained by women’s closer engagement with healthcare services and providers. This connection is often established through prenatal care; childbirth assistance; child healthcare; and the care of other family members, including the elderly [20,21].

More than half of the interviewees reported having experienced mental health issues, such as depression, sadness, unexplained crying, and excessive stress. While this study did not aim to delve into the causes, it is noteworthy that the population studied also showed a predominance of overweight and obesity. In recent years, the connection between obesity and mental health disorders has gained particular attention. It has been observed that the prevalence of obesity may be up to three times higher in individuals with mental health disorders. Conversely, obesity has been identified as an independent predictor of several mental health disorders, particularly depression and anxiety [22].

Despite strong evidence for the health risks associated with the frequent consumption of energy-dense foods, high in fat and added sugars, these foods remain widely preferred due to their palatability and early-life exposure, serving as powerful sources of neurobiological reward [23]. The key factors influencing food preferences include familiarity, sweetness, and energy density, with repeated exposure further shaping these preferences [22,24]. Clinical studies have suggested that cravings are most directed toward foods that are high in fat, sugar, or both. Historically, in times of dietary scarcity, a preference for energy-dense foods conferred a survival advantage [25]. Given these factors, it is crucial to consider them when designing counseling strategies and educational programs in primary healthcare settings. Extending these efforts to schools could also help instill healthier eating habits from an early age.

Targeted interventions to improve obesity rates among populations living in vulnerable communities were also recommended in a recent report from the PAHO and WHO, emphasizing the critical importance of reducing unhealthy diets and improving physical activity and sleep quality [7]. A systematic review [20] further highlighted the importance of market-based food interventions, including, but not limited to, higher taxes on processed foods and beverages with a high sugar content and nutritional labeling on packaged foods to address the growing obesity levels in Latin American countries.

Finally, while fruit and vegetable production in rural Nicaragua is a critical component of the country’s agricultural sector and contributes significantly to both food security and the livelihoods of small-scale farmers, the consumption of healthy food, such as fruits and vegetables, among rural residents is considered weak or non-existent in their regular diet. This is contradictory, as rural areas have more access to traditional and organic foods. Choosing a diet plan is generally a complex process that may be influenced by family, mass media, and the environment. In addition, globalization can affect people’s preferences for traditional food while providing a wider choice and availability of food; therefore, it is also a factor that influences people’s dietary choices [26].

These results may also be influenced by the accessibility of information that enables people to easily obtain the knowledge they seek, particularly regarding the nutritional aspects of food. Rapid technological advancements have significantly transformed lives across all regions, offering modern conveniences and lifestyle choices that have often replaced traditional practices. Social media can foster either positive or negative habits, depending on the quality and reliability of the information sources [27]. It is widely recognized as an accessible, cost-effective, and culturally appropriate platform that ensures equal access to information while also serving as an engaging and trusted resource, particularly in rural communities. With the continuous expansion of social media platforms, there is great potential to encourage healthy habits from an early age. However, many sponsored advertisements on these platforms promote fast food, high-fat meals, and excessive carbohydrate consumption, which can contribute to unhealthy lifestyle choices.

Another relevant factor to consider is the low educational level among our participants. Previous studies have shown that favorable attitudes toward healthy eating are associated with both higher educational levels and diet quality. Our results suggest that poor attitudes toward healthy eating in groups with low socioeconomic status constitute an additional factor (along with cost constraints) in the choice of unhealthy foods [28].

Our results show that the respondents who reported weak or no consumption of vegetables tended to have a higher waist–hip ratio compared to their counterparts. Based on a review of experimental data, researchers have suggested that the foods found in vegetarian diets may have metabolic advantages for the prevention of type 2 diabetes [21], and that fruits and vegetables are associated with a 40% reduction in type 2 diabetes [29,30,31,32,33].

In general, the present study has several strengths, including the recommended use of the WHR as a more accurate indicator of body fat distribution and its association with health risks like mortality and morbidity, particularly in Hispanic and Latino populations; it highlights the significant gender disparities in obesity prevalence, emphasizing both biological and socioeconomic factors; by analyzing the respondents’ self-perceived health status, sleep patterns, dietary habits, and work conditions, this study provides a comprehensive view of the behaviors contributing to obesity in rural Nicaragua; and it paves the way for actionable recommendations, such as promoting vegetable consumption, enhancing sleep quality, and creating culturally tailored nutritional education initiatives. These interventions align with the PAHO and WHO reports, adding credibility and practicality.

This study has several limitations worth noting. Firstly, the cross-sectional design and the fact that the data were gathered from only three rural communities within a single department in Nicaragua restrict the generalizability of the findings. However, since the characteristics of the rural population are somewhat comparable across the country, we are confident that our study results are very generalizable to the rural communities in Nicaragua. Secondly, cultural practices and the stigma associated with mental health and food intake may have influenced individual responses to the related questions. We advise readers to consider these factors.

## 5. Conclusions

Despite significant and notable improvements in Nicaragua’s health sector in recent years, the rise in non-communicable diseases among the most vulnerable populations, particularly those in rural areas, poses a major threat to the public health system. The waist–hip ratio (WHR) is a better indicator of mortality compared to the BMI, especially for Hispanic and Latino populations. The results presented in this study are unique in using the waist–hip ratio (WHR), contributing further to our understanding of the growing public health concerns regarding overweight and obesity in Nicaragua. Massive healthy eating campaigns are needed in rural areas, as the identified eating habits are significant predictors of a high waist–hip ratio (WHR), which is clearly a risk factor for cardiovascular morbidity and mortality from chronic diseases. These findings should be considered in the development of health policies and interventions specifically tailored to rural populations in the country, with the aim of promoting the selection, preparation, and consumption of fruits and vegetables, as well as increasing physical activity, as strategies for improving individuals’ quality of life and reducing the burden of obesity and cardiovascular disease.

## Figures and Tables

**Table 1:** General characteristics and self-perceived health.

Background Characteristics of the Respondents (*n* = 200)		
Characteristics	*n*	Percentage
Age		
30–34	47	23.5
35–39	24	12.0
40–44	23	11.5
45–49	28	14.0
50–54	22	11.0
55–59	20	10.0
60–64	14	7.0
65+	22	11.0
Gender		
Male	45	22.5
Female	155	77.5
Educational level		
Primary	98	49.0
Secondary	79	39.5
Professional	14	7.0
No education	9	4.5
Self-reported health		
Excellent	1	0.5
Very good	9	4.5
Good	54	27.0
Fair	135	67.5
Poor	1	0.5
Mental health issues in the last 30 days		
Never	97	48.5
Less than 3 days	12	6.0
3–7 days	8	4.0
8+ days	83	41.5
Has a health professional ever told you have		
High blood pressure	57	28.5
Diabetes	18	9.0
Depression	1	0.5

**Table 2:** Behavioral and clinical characteristics of the respondents (*n* = 200).

Characteristics	*n*	Percentage
Last routine medical check-up		
Less than 1 year	151	75.5
1–2 years ago	43	21.5
More than 2 years ago	4	2.0
Never	2	1.0
Smoking status		
Yes	7	3.5
Use of alcohol		
Yes	10	5.0
Vegetable consumption		
Once a week	41	20.5
Two or more times a week	25	12.5
Never	134	67.0
Salad consumption		
Once a week	9	4.5
Two or more times a week	59	29.5
Never	132	66.0
Hours of sleep per day (mean, Std. dev.)	200	(7.3, 1.46)
BMI		
Less than 25	33	16.5
25.0–29.9	84	42.0
30.0+	83	41.5
Blood pressure		
Normal	147	73.5
Elevated	36	18.0
High grade 1	15	7.5
High grade 2	2	1.0
Glycemia level		
Normal	152	76.0
Above normal	48	24.0
Waist–hip ratio		
Low	18	9.0
Moderate	22	11.0
High	160	80.0

**Table 3:** Bivariate distribution of health characteristics of the respondents for background and behavioral characteristics.

Variables	Waist–Hip Ratio		Sig.
	Low/Moderate	High	
Age			*p* < 0.05
<40	28.2	71.8	
40+	15.5	84.5	
Gender			*p* < 0.05
Male	35.6	64.4	
Female	15.5	84.5	
Marital status			ns
Living alone	20.6	79.4	
Living with someone	19.7	80.3	
Education level			ns
Primary or less	17.8	82.2	
More than primary	22.6	77.4	
Self-reported health			ns
Good or better	21.9	78.1	
Fair or poor	19.1	80.9	
Hours of sleep			*p* < 0.05
7 h or less per day	26.7	73.3	
More than 7 h	17.1	82.9	
Vegetable consumption			*p* < 0.001
Once a week or never	12.8	87.2	
2 or more times a week	37.3	62.7	

ns—Not significant.

**Table 4:** Odds of predicting high waist–hip ratio in rural Nicaragua (*n* = 200).

	Model 1		Model 2	
Variables	AOR	Sig.	AOR	Sig.
Age				*p* < 0.05
<40			1.00	
40+			2.76	
Gender				*p* < 0.001
Male			1.00	
Female			3.58	
Marital status				ns
Living alone			1.00	
Living with someone			1.37	
Educational level				ns
Primary or less			1.00	
More than primary			0.95	
Self-reported health		ns		ns
Regular or poor	1.00		1.00	
Good or better	1.27		1.70	
Hours of sleep		*p* < 0.05		*p* < 0.05
7 h or less	1.00		1.00	
7+ h	2.38		2.07	
Veg. consumption		*p* < 0.001		*p* < 0.001
No veg.	1.00		1.00	
2+ times a week	0.20		0.21	
−2 Log likelihood	180.93		168.39	

Model 1 is adjusted for behavioral variables, such as self-reported health, hours of sleep, and vegetable consumption. Model 2 includes both behavioral variables and background characteristics (age, gender, marital status, and educational level). ns: Not significant.

## Data Availability

The raw data supporting the conclusions of this article will be made available by the authors on request.
